# Biomolecular Study of the Correlation Between Papillomatosis of the Vulvar Vestibule in Adolescents and Human Papillomavirus

**DOI:** 10.1100/tsw.2006.130

**Published:** 2006-06-12

**Authors:** Geni Beznos, Veronica Coates, Jose Focchi, Hatim A. Omar

**Affiliations:** ^1^ Adolescent Clinical Unit, Department of Pediatrics, Irmandade Santa Casa de Misericórdia de São Paulo, Santa Casa de São Paulo, Faculty of Medical Sciences, São Paulo, Brazil; ^2^ Department of Pediatrics, University of Kentucky, Lexington, USA

**Keywords:** HPV, papillomatosis, adolescents, Brazil, United States

## Abstract

The main goal of this study was to investigate, through a biomolecular study, the correlation between papillomatosis of the vulvar vestibule and human papillomavirus (HPV) infection, as well as to establish the necessity of treatment. A total of 44 female adolescents between 12 and 18 years of age were selected through a prospective study with a confirmed diagnosis of papillomatosis of the vulvar vestibule. Vulvar biopsies were obtained for the histological and biomolecular detection of HPV DNA through polymerase chain reaction (PCR). Twenty (45%) adolescents were virgins (group A), the other 24 (55%) were sexually active. The virgin adolescents (group A) and 12 sexually active adolescents (group B) did not show cytological and/or colposcopic alteration, suggesting infection by HPV either on the cervix or vagina. These were compared with 12 other sexually active adolescents who showed cervicovaginal infection caused by HPV (group C). Fisher exact test was applied for statistical analysis of the results, considering alpha equal or less than 0.05. There was no statistically significant difference in relation to HPV DNA through PCR among virgin and sexually active adolescents in group B, however, both differed from those in group C (A + B × C: *p* = 0.048*). The histological study did not reveal evident signs of infection caused by HPV on vestibular papillae, besides perinuclear halos. HPV DNA was detected on vestibular papillae in 27%. Our results confirmed a scarce correlation between vestibular papillae and HPV. Thus, we consider papillomatosis of the vulvar vestibule, in most cases, to be equivalent to physiological papillomatosis and, therefore, should not be treated.

## INTRODUCTION

Genital human papillomavirus (HPV) infection is one of the most common sexually transmitted infections in sexually active adolescent and young adult women[1]. HPV 6 and 11 are largely responsible for benign warts, while types 16 and 18 are primarily associated with intraepithelial neoplasias and invasive carcinomas[2]. The diagnosis of anogenital warts does not present significant difficulties. The acuminate warts are easily recognized with their numerous superficial filiform projections that coalesce in a common base, densely veined and sessile, forming extensive and voluminous vegetations, which assume the appearance of cauliflower. Conversely, the diagnosis of the vulvar subclinical forms is more complex due to greater number of physiological and pathological conditions that may appear similar[3].

In 1987, Campion[4] described three types of subclinical vulvar lesions, one of which was the vestibular papillae, and their relationship to the HPV infection. The report by the International Society for the Study of Vulvar Diseases (ISSVD)[5] considered papillomatosis of the vulvar vestibule as the presence of multiple papillae that may cover the mucosal surface of the labia minora; it also reported that the visualization of papillomatosis and parakeratotic changes may be facilitated by *acetowhitening*, the result of applying a 3–5% acetic acid solution (vinegar) to the epithelium for 1–2 min. Papillomatosis is a relatively common clinical finding, however, its origin is yet uncertain.

Papillomatosis of the vulvar vestibule has been seen with HPV infection, but a consistent association has not been proven. There is controversy as to the etiology in the descriptions made by several authors[6,7,8,9,10,11,12,13,14,15,16].

Some authors have reported that papillomatosis of the vulvar vestibule is a normal variant in the vestibular architecture not related to infection by HPV[6,9,15,16,17,18,19,20]. Others[2,7,8,10,21,22, 23,24,25] have reported that papillomatosis of the vulvar vestibule is a consequence of HPV infection. However, it has not been demonstrated convincingly that HPV plays a clear role in the pathogenesis of papillomatosis of the vulvar vestibule. The frequent finding of papillomatosis in the vulvar vestibule in virgin adolescents and its controversy in the literature raised doubts as to its etiology and motivated this study. The purpose of the present study was to verify, through a biomolecular study, the correlation between papillomatosis of the vulvar vestibule in virgin and nonvirgin adolescents and infection by HPV, as well as the necessity or not of treatment.

## METHODS

Through a prospective study, 44 female adolescents between ages 12 and 18 years, with a diagnosis of papillomatosis of the vulvar vestibule, were recruited for the study at the Adolescent Clinic of the Pediatric Department of Santa Casa of São Paulo, Brazil.

Papillomatosis of the vulvar vestibule in all of the adolescents was visible to the naked eye and its diagnosis was confirmed by vulvoscopy. Numerous digitiform projections with a single base and central vascular axis, symetrically distributed on the internal walls of the labia minora, rosy and soft, were observed in all patients (see Fig. 1). Of the 44 adolescents, 20 (45%) were virgins and denied any intimate sexual contact, prior history of genital tract condilomata, or sexual abuse (group A). They did not show any cytological and/or colposcopic alterations, suggesting infection by HPV in the cervix or vagina. The hymeneal integrity was confirmed by clinical examination.

The other 24 (55%), sexually active adolescents, were subdivided into two groups. Group B was composed of 12 adolescents who, besides papillomatosis of the vulvar vestibule, did not present current evidence of HPV infection or neoplasia, nor colposcopic alterations suggestive of HPV infection in the cervix or vagina in the cervicovaginal cytologic study. They also did not present a prior history of genital tract condilomata and/or cervical dysphasia. Group C was composed of 12 adolescents who, along with the papillomatosis of the vulvar vestibule, presented alterations suggestive of cervicovaginal infection by HPV, detected by cervical Papanicolaou smear, histology, and/or colposcopy.

The age of the virgins varied between 12 and 17 years, the average being 14.5 years, whereas the nonvirgins belonged to the age range of 14 to 18, with an average of 16 years. The average age at first sexual intercourse among the sexually active was 15 years — 23 (96%) reported a single sexual partner and only 1 (4%) reported two; 27 (62%) of the total study population were white, 12 (27%) were of mixed race, and 5 (11%) were black; 35 (80%) were asymptomatic and 9 (20%) presented with symptoms such as itching and vulvar burning.

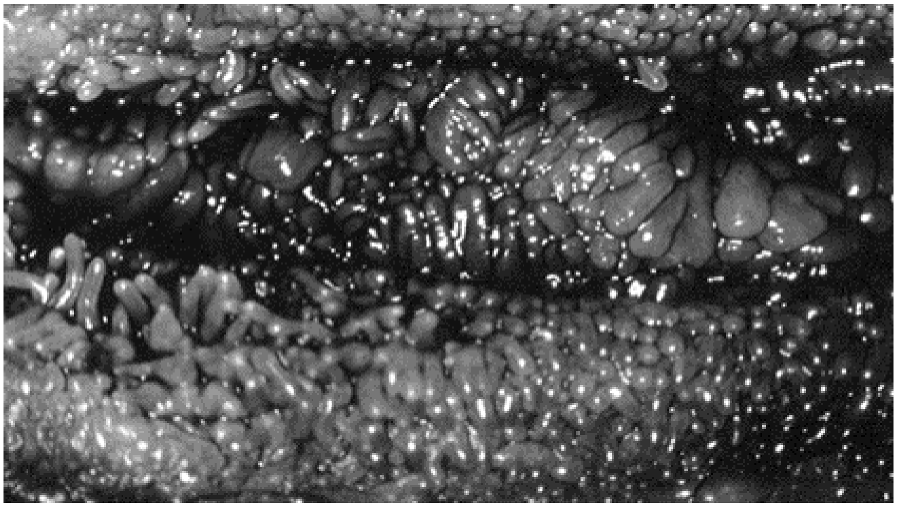

**FIGURE 1**. Typical appearance of vulvar vestibular papillomatosis.

During colposcopic evaluation, vulvar biopsy was performed on areas affected by papillomatosis, both for histologic examination and for HPV DNA determination by polymerase chain reaction [PCR] technique. The biopsy was obtained with a 6-mm punch from the inner surface of the labia minora of each patient in the three groups. The vulvoscopic and colposcopic examinations, the vulvar biopsy, and the anatomopathologic study were performed at the Oncocentro Foundation of São Paulo, always by the same professional and conveniently preserved material. The material obtained from the biopsy was fixed in 10% buffered formalin, embedded in paraffin, and histologic slices of 7 μm were stained in a routine manner with hematoxylin and eosin, and examined under a common light microscope. The paraffin block was sent for DNA extraction to the Ludwig Institute of São Paulo, which was performed according to publications of Pinto and Villa in 1998[26]. In the PCR technique, genic amplification was obtained with the primers GP5+ and GP6+, capable of amplifying many HPV types, using the method described by Snijders et al.[27]. Positive and negative DNA controls were used to interpret the results.

The study was analyzed and approved by the Medical Ethics Committee of Oncocentro Foundation of São Paulo. Written consent was given by all of the patients and/or their legal guardians in order to perform the procedures. Data were analyzed statistically by Fisher exact test, with the level of significance set at $p < 0.05$. When the calculated statistic presented significance, an asterisk [*] was used to identify it.

## RESULTS

There was a discreet enhancement of the micropapilar salience following the application of 5% acetic acid solution and a faint impregnation by iodine from the Collins test in all of the patients. The 44 (100%) biopsy samples of papillomatosis of the vulvar vestibule were histologically characterized as papillas formed by the conjunctive-central vascular axis and coated with squamous epithelium. Koilocytosis was observed in the 44 (100%) samples of vestibular papillae analyzed. Papillomatosis, acanthosis, and occasionally parakeratosis were also observed.

By means of the molecular biology technique, 4 (20%) of the 20 samples of vestibular papillae analyzed in group A tested positive for HPV by PCR. In group B, of the 12 samples of vestibular papillae analyzed, 2 (17%) tested positive for HPV and of the 12 samples analyzed from group C, 6 (50%) tested positive for HPV by PCR.

Comparing groups (A + B), which were not statistically different from one another (see Table 1), to group C in relation to the positivity for HPV by PCR, a statistically significant difference was observed ($p = 0.048$) less than the stipulated critical value (see Table 2).

**TABLE 1**
**Presence of HPV DNA in Biopsied Fragment of the Vestibular Papillomatosis of Group A and of Group B from the PCR and Statistical Results**

| GROUP/HPV | YES | NO | TOTAL |
|---|---|---|---|
| A | 4 | 16 | 20 |
| B | 2 | 10 | 12 |
| Total | 6 | 26 | 32 |

Fisher Exact Test

*p* = 0.60 (unicaudal)

**TABLE 2**
**Presence of HPV DNA in Biopsied Fragment of Vestibular Papillomatosis in Groups A and B vs. Group C from the PCR and Statistical results**

| GROUP/HPV | YES | NO | TOTAL | % YES | % NO |
|---|---|---|---|---|---|
| A + B | 6 | 26 | 32 | 18.75 | 81.25 |
| C | 6 | 6 | 12 | 50 | 50 |
| Total | 12 | 32 | 44 | 27.27 | 72.73 |

Fisher Exact Test

A + B × C: *p* = 0.048* or 4.80%*

## DISCUSSION

Papillomatosis of the vulvar vestibule has been a subject of controversy as to its nomenclature, etiopathogeny, clinical and/or vulvoscopic aspect, clinical findings, and necessity or not for treatment. In the literature, a variety of terminologies may be found, which reflects the uncertainty as to its etiology, including (among others) pseudocondylomata of the vulva[6], vestibular papillae[9,17], pruritic vulvar squamous papillomatosis[7,22], hirsutoid papillomatosis of the vulva[18,28], micropapillomatosis labialis[29], squamous papillomatosis[10], and vestibular papillomatosis[2,11,13,25,30,31].

Various clinical and vulvoscopic descriptions of these papillar projections are found in the literature as to its prominence and general appearance in the vestibular tissue, which may raise doubts concerning the interpretation. In this study, care was taken to select only those patients who were diagnosed as having papillomatosis of the vulvar vestibule according to the report by the ISSVD.

In relation to the histological aspects, we verified in the literature that even experienced pathologists encountered difficulty in distinguishing the reaction of the vulvar epithelium induced by vírus from that of a normal vulvar mucosa. This fact was well established in a study done by Spitzer et al. in 1990[32], employing five pathologists, histologically comparing biopsies of the cervix and the vulva with hybridization *in situ* of 21 patients with altered Pap smear results. They observed that in the uterine cervix, the results were strongly related to the biomolecular result, whereas in the vulva, there was a lack of agreement among the pathologists, in disagreement on all of the criteria, with the exception of papillomatosis. They justified this by the fact that the vulva presents a greater degree of differentiation than the uterine cervix and because of the number of glycogenated cells present in the vulvar epithelium. The classic koilocytosis may be considered pathognomonic of infection by HPV, according to Abrão et al. in 1994[33], but it is of fundamental importance that this term be utilized for the cells that present significant nuclear alterations secondary to the presence of viral DNA in the nucleus, such as hypercromasia, irregular nuclear contour, cariomegalia, and multinucleation, simultaneously with cytoplasmatic alterations, such as the anomalous cytoplasmatic keratinization.

Bergeron et al. in 1990[29], on the histological analysis of 34 patients with vestibular papillae, verified the presence of papillomatosis, acanthosis, and sometimes parakeratosis in all samples, but which lacked the classic koilocytes, that is, those with atypical cytology, multinucleated cells, or dyskeratotic cells. The authors point out the presence of glycogenated cells, which can be misinterpreted as koilocytes.

In our study, the presence of koilocytosis was observed in the 44 (100%) patients with a diagnosis of vestibular papillomatosis. However, koilocytotic changes suggestive of viral infection by HPV (classic koilocytosis) were not observed. The HPV DNA was detected in 27% of the cases, demonstrating in this manner a lack of consistent correlation between the molecular biology diagnosis and the histologic findings.

Various diagnostic techniques were used in the literature to clarify the etiology of the papillomatosis of the vulvar vestibule, and contradictory results were observed. The reason for such findings may have been due to the utilization of different methods of sensitivities and the sample collection technique. In the study performed by de Deus[19], on comparing patients who were papillomatosis of the vulvar vestibule carriers and others who presented condyloma acuminatum, and still others with normal vulvar epithelium, the author used only patients with normal specular, cytologic, and colposcopic examinations as a criteria for inclusion in the study in the papillomatosis of the vulvar vestibule group, aiming to exclude the possiblility that the vulvar epithelium might be contaminated with HPV of cervicovaginal origin. With these more rigorous criteria adopted by the author, the HPV DNA was observed in only 4% of the papillomatosis of the vulvar vestibule cases by the dot blot molecular hybridization and in 6.67% of the cases by the PCR.

In our study, we were careful to select some patients who did not have a history of genital HPV infection, with the objective of excluding the possibility of vulvar contamination. We were also careful to subdivide the sexually active patients into two groups: those without and those with alterations suggestive of cervicovaginal HPV infection (groups B and C, respectively) in order to evaluate with this criterion the real value of the presence of HPV in the vulva. Utilizing as criterion the absence of cytologic and/or colposcopic alterations suggestive of cervical or vaginal HPV infection, the HPV DNA was observed with the PCR in four (20%) of the patients in group A, in two (17%) of the patients in group B, and, without the use of this criterion, the HPV DNA was observed in six (50%) of the patients in group C. We also observed in our study that there was no statistically significant difference between the group of adolescent virgins and the group of sexually active adolescents in group B, as to the positivity of HPV with the PCR, when this criterion was employed. On the other hand, when both groups were compared to the group of sexually active patients in group C, we verified a statistically significant difference as to the presence of HPV in the vulva, demonstrating in this way the lack of consistent association between the papillomatosis of the vulvar vestibule and the presence of HPV when selection criterion like the one we utilized is adopted.

The use of this criterion was not observed in many of the publications that associated papillomatosis of the vulvar vestibule with HPV[2,7,21,25,34,35], in spite of the multifocal and multicentric nature of this infection being clear in the literature. According to the literature, there was no doubt that the principal means of transmission of genital infection by HPV in adolescents occurs through sexual contact. However, the finding of HPV DNA in 20% of the adolescent virgins in our study agrees with the literature in that the finding of HPV DNA observed in young women who deny intimate sexual contact or relations suggests that other means of transmission besides the sexual one may be possible.

In relation to the adolescents, in spite of having little available data on the prevalence of HPV DNA, isolated studies demonstrated rates that varied from 0–30% in adolescents who were not sexually active and from 13–38% in sexually active adolescents[36].

A study performed by Bergeron et al.[29] detected HPV DNA in the undergarments of patients with anogenital HPV infection. The HPV types 6/11 were observed in 69% and types 16, 18, 30, 31, 33, and 35 in 31%. The authors concluded that the undergarments of patients with anogenital HPV infection might have been contaminated. They pointed out the importance of this fact for the adolescents who have the habit of exchanging clothes with their friends.

Pacheco et al.[37] verified clinical and subclinical lesions by HPV infection in 75%, and histologically confirmed in 62% of the cases in young virgins (under 18 years of age), daughters and sisters of 31 adults, who presented anogenital warts. In this same study, the authors verified in 10 young virgins (under 18 years of age) who presented with anogenital warts, the presence in 90% of the cases of one or more members of the family infected by HPV. The authors also observed that in the majority of cases there was presence in the family of one or more of the following practices: common use of towels, bathrooms, bidets, shared use of the same bed, undergarments, and swimsuits.

Pao et al.[38], compared the vulvar biopsies of one group of young virgins with those of a group of sexually active youths, but did not observe a statistically significant difference between the groups as to the prevalence of HPV DNA with PCR. HPV types 6, 16, and 18 were detected in both groups. The authors suggested other means of infection transmission besides the sexual one. Wheeler et al.[39] observed the presence of HPV DNA with PCR in 2 (15.4%) of the cervical samples of 13 young women who stated they had never had any sexual experience. These studies raise important ethical considerations, as asymptomatic HPV infections are not uncommon in youths who have never had any former sexual experience, suggesting that in fact the infection may be acquired through transmission means other than sexual.

Similar to our study, Pao et al.[13] observed papillomatosis of the vulvar vestibule in adolescents who denied any intimate sexual contact (average age of 17 years). According to these authors, the finding of these papillar projections in adolescent virgins suggests they were physiologic.

We believe that the finding of 20% of the HPV DNA in the adolescent virgins in our study may be related to contamination by other means, which was not sexual and that the infection was encountered in its latent form, being related to prevalence of HPV DNA in the healthy population. The results of our study did not give us support to confirm the viral origin in the diagnosis of vestibular papillae, since the histologic results did not demonstrate evidence of clear infection by HPV of the papillae. Utilizating of molecular biology technique, HPV DNA was detected in only 12 (27%) of the total number of samples of biopsied papillomatosis of the vulvar vestibule, while the HPV DNA was encountered in condyloma acuminatum in 100% of the cases[40].

Finally, when a more rigid criterion for inclusion was used, such as that for groups A and B to preclude the possibility of contamination, we observed a statistically significant difference in relation to the presence of HPV DNA in the vulva than when this criterion was not utilized. The finding of 27% of HPV DNA in this study therefore included the presence of a group of adolescents with HPV infection in the cervix and/or vagina (group C), with a greater probability of contamination of the vulvar epithelium, thus being responsible for the increase in our study, which would decrease to 18% (6/32) in case this group was excluded. The prevalence of HPV DNA found in our study may represent the frequency in which the HPV DNA was found in normal mucosa in its latent form.

In light of what was demonstrated, we conclude that papillomatosis of the vulvar vestibule was not consistently related to HPV and cannot be diagnosed exclusively on either the basis of presence of clear perinuclear haloes or presence of faint aceto-white reactions in vulvoscopy. We also conclude that biopsy of the vestibular papillae should be avoided due to diagnostic difficulty frequently observed in this region of the vulva, and being unnecessary in light of the utilization of molecular biology technique as routine procedure. However, we deem the role of vulvoscopic evaluation for precise diagnosis in doubtful cases to be fundamentally important.

We consider papillomatosis of the vulvar vestibule as a normal variant in the vulvar vestibule architecture, not directly related to infection by the HPV in agreement with others[6,9,15,16,17,18,19,20]. Based on our findings, we believe that papillomatosis of the vulvar vestibule should not be treated, avoiding unnecessary procedures in this way, as well as harmful emotional reactions on part of the patients, many still virgins, who may erroneously presume themselves infected by a neoplasm-inducing and sexually transmissible virus. However, those patients who, in addition to papillomatosis of the vulvar vestibule, also present other HPV infection focci in the cervix and/or vagina should receive adequate treatment in these sites, being important rigorous clinical observation of the vulvar region, as these are patients at risk to develop the infectant form and lesions, which are precursors of vulvar intraepithelial neoplasia.

## ACKNOWLEDGMENTS

We are grateful to the Support Center for Scientific Publications of Santa Casa de São Paulo, Faculty of Medical Sciences for the editorial assistance. We thank Luiza Villa for excellent technical assistance.

## BIOSKETCHES

**Geni W. Beznos, MD,** Medical Assistant**,** Adolescent Clinic, Department of Pediatrics, Irmandade da Santa Casa de Misericórdia de São Paulo, Santa Casa de São Paulo, Faculty of Medical Sciences, São Paulo, Brazil. E-mail: genibeznos@terra.com.br

**Veronica Coates**, **MD**, Professor, Adolescent Clinic and Bone Metabolism Unit, Department of Pediatrics, Irmandade da Santa Casa de Misericórdia de São Paulo, Santa Casa de São Paulo, Faculty of Medical Sciences, São Paulo, Brazil. E-mail: veronicacoates@attglobal.net

**José Focchi, MD,** Associated Professor in Gynecology and Obstetrics**,** Department of Gynecology and Obstetrics UNIFESP, Faculty of Medical Sciences, São Paulo, Brazil. E-mail**:** jfocchi@aol.com

**Hatim A. Omar, MD**, Professor of Pediatrics, Obstetrics and Gynecology and Director of the Section of Adolescent Medicine, Department of Pediatrics, University of Kentucky, Lexington. He is the recipient of the Commonwealth of Kentucky Governor's Award for Community Service and Volunteerism. He is an expert in adolescent medicine and pediatric and adolescent gynecology. E-mail: haomar2@uky.edu
